# Learning effectiveness of a flexible learning study programme in a blended learning design: why are some courses more effective than others?

**DOI:** 10.1186/s41239-022-00379-x

**Published:** 2023-02-17

**Authors:** Claude Müller, Thoralf Mildenberger, Daniel Steingruber

**Affiliations:** Zurich University of Applied Sciences, St. Georgenplatz 2, 8401 Winterthur, Switzerland

**Keywords:** Blended learning, Flexible learning, Learning effectiveness, Higher education, Educational design

## Abstract

Flexible learning addresses students’ needs for more flexibility and autonomy in shaping their learning process, and is often realised through online technologies in a blended learning design. While higher education institutions are increasingly considering replacing classroom time and offering more blended learning, current research is limited regarding its effectiveness and modifying design factors. This study analysed a flexible study programme with 133 courses in a blended learning design in different disciplines over more than 4 years with a mixed-methods approach. In the analysed flexible study programme, classroom instruction time was reduced by 51% and replaced with an online learning environment in a blended learning format (*N* students = 278). Student achievement was compared to the conventional study format (*N* students = 1068). The estimated summary effect size for the 133 blended learning courses analysed was close to, but not significantly different from, zero (*d* = − 0.0562, *p* = 0.3684). Although overall effectiveness was equivalent to the conventional study format, considerable variance in the effect sizes between the courses was observed. Based on the relative effect sizes of the courses and data from detailed analyses and surveys, heterogeneity can be explained by differences in the implementation quality of the educational design factors. Our results indicate that when implementing flexible study programmes in a blended learning design, particular attention should be paid to the following educational design principles: adequate course structure and guidance for students, activating learning tasks, stimulating interaction and social presence of teachers, and timely feedback on learning process and outcomes.

## Introduction

Considering the digitalisation of society, there is an increasing need to constantly develop one’s competencies in the sense of continuous lifelong learning (OECD, 2019). In this context, higher education should be adapted to the learners' diverse needs and specific live phases (Barnett, 2014; Martin & Godonoga, 2020) and accessible to broader sections of the population (Dziuban et al., 2018; Orr et al., 2020). The concept of flexible learning addresses these needs and tries to afford learners more flexibility and autonomy in shaping the learning process regarding when, where, and how they learn (Boer & Collis, 2005; Hrastinski, 2019; Lockee & Clark-Stallkamp, 2022; Smith & Hill, 2019; Vanslambrouck et al., 2018; Wade, 1994). From a pedagogical point of view, different dimensions of flexible learning can be distinguished. Li and Wong (2018) analysed previous publications and identified the following dimensions of flexible learning—time, content, entry requirement, delivery, instructional approach, performance assessment, resources and support, and orientation or goal. The frequently mentioned dimension of place (e.g. Chen, 2003) belongs in this concept to the delivery dimension. By designing the above dimensions according to learners' needs, the students should actually perceive learning as flexible. From a technical perspective, flexible learning has often been attempted through online technologies (Tucker & Morris, 2012). According to Allen et al. (2007) learning environments can be classified according to their proportion of online content delivery either as traditional with no online delivery content, as web-facilitated (with an online delivery proportion of between 1 and 29 per cent), blended learning (with an online delivery proportion of between 30 and 79 per cent) or online learning with more than 80 per cent of online delivery content. Accordingly, flexible learning is often associated and used in connection with blended or online learning (Anthony et al., 2020).

The COVID-19 pandemic, with its global shift to remote instruction, has accelerated the demand for flexible learning options in higher education (Lockee & Clark-Stallkamp, 2022; Pelletier et al., 2022). Current student evaluations have shown that the experienced learning flexibility during ‘emergency distance learning’ (Hodges et al., 2020) is appreciated (Gherheș et al., 2021; Shim & Lee, 2020) and students are demanding more flexible learning options in the aftermath of the pandemic as well (Clary et al., 2022; Lockee & Clark-Stallkamp, 2022). In response, higher education institutions are now considering replacing classroom time and offering more online and blended learning formats (Kim, 2020; Pelletier et al., 2021; Peters et al., 2020; Saichaie, 2020).

Despite the apparent popularity of blended learning, academics are often concerned about the effectiveness of blending for student learning (Huang et al., 2021), and educational institutions will only be able to offer and expand blended learning formats when they are confident that students will perform as they would in a conventional classroom setting (Owston & York, 2018). Meta-analyses (Bernard et al., 2014; Means et al., 2013; Müller & Mildenberger, 2021; Vo et al., 2017) point out that blended learning is not systematically more or less effective than conventional classroom learning. At the same time, they have pointed out that the number of controlled studies is still limited and that the studies have examined mostly single courses with a study period of one semester; there is a particular lack of controlled studies at a degree level (i.e., with many courses taught over a longer period). In addition, variance in the learning effectiveness of the courses found in the studies was large, with a shortage of studies on the implementation and design success factors of blended learning based on objective learning achievement rather than student and lecturer evaluation (Bernard et al., 2019; Graham, 2019; Means et al., 2014).

### Research questions

This study addressed the above issues of learning effectiveness and modifying factors of blended learning at the study and course levels. The focus of the researched study programme was to give students more flexibility in the learning process, especially regarding time and place, by replacing classroom time with an online learning environment in a blended learning design (see details in the research context). Accordingly, the term ‘flexible learning’ is used in this paper as desired study characteristics at the programme level. The term ‘blended learning’ is used to describe the educational design of the courses under investigation in the experimental condition.

The two research questions (RQ) were:

1. What is the impact on *student achievement* (measured as exam results) of blended learning with classroom time reduced by half at the course level and study programme level in a flexible learning study programme compared with the conventional study format?
2. What are the *modifying factors* for the learning effectiveness of blended learning courses with reduced classroom time in a flexible learning study programme?

## Literature review

### Student achievement

Several studies have explored the acceptance and effectiveness of blended or online environments with reduced classroom time in recent years. In a study by Asarta and Schmidt (2015), presence in classroom sessions in a traditional course was compared with an experimental setting where lectures were also made available online. In the two settings, the exams, learning materials, and number of planned classroom sessions were identical, but students could choose whether to attend classroom sessions in the blended learning version. Data analysis showed that students reduced their average attendance to between 49 and 63%. Asarta and Schmidt (2015) concluded that—in line with the student preferences—the classroom attendance rate in blended learning courses could be reduced by approximately one-half compared with conventional courses. This is one of few studies in which students had control over the blend ratio; usually, the instructor decides and takes responsibility for the proportion of instruction delivered in a blended learning format (Boelens et al., 2017).

Owston and York (2018) investigated the relationship between the proportion of online time spent in blended learning courses and student satisfaction and performance. The clustering was determined by the ratio of time spent on online activities replacing classroom sessions. The results showed that students in courses with high (50%) and medium (between 36 and 50%) online proportions rated their learning environments more positively and performed significantly better than their peers in blended learning courses with low (27–30%) or supplemental online segments. Consequently, Owston and York (2018) concluded that across a wide variety of subject areas and course levels, student perceptions and performance appeared to be higher when at least one-third to one-half of regular classroom time was replaced with online activities.

Hilliard and Stewart (2019) came to similar conclusions concerning satisfaction. They examined the student perceptions of the various aspects of the community of inquiry (COI) model, and their findings indicated that students in high blend (50% online) classes perceived higher levels of teaching, social, and cognitive presence than students in medium blend (33% online) classes.

In a recent review, Müller and Mildenberger (2021) examined the impact of replacing classroom time with an online learning environment. Their meta-analysis of blended learning (*k* = 21 effect sizes) applied strict inclusion criteria concerning research design, learning outcomes measurement, and blended learning implementation. In particular, it was a requirement that the attendance time in the blended learning format was reduced by 30–79% compared to the conventional learning environment, drawing on Allen et al. (2007). In this meta-analysis, the estimated effect size (Hedge’s g) was 0.0621, although not significantly different from zero. The confidence interval [lower 95th − 0.13, upper 95th 0.25] suggests that overall differences between blended and conventional classroom learning were small, and, at best, very small negative or moderate positive effects were plausible. This implies that despite a reduction in classroom time of between 30 and 79 per cent, equivalent learning outcomes were found. However—in line with authors of other blended learning reviews (Bernard et al., 2014; Means et al., 2013; Spanjers et al., 2015; Vo et al., 2017)—it was pointed out that the number of controlled studies in the field of blended learning was still limited. More primary studies of the highest methodological quality must be conducted in various disciplines to validate the results further and investigate the effectiveness of blended learning in different disciplines and contexts. Additionally, the authors emphasised considerable heterogeneity in the effect sizes between the various studies. McKenna et al. (2020) also stated that simply offering a blended learning course is not enough to ensure success; research on blended learning design should, therefore, differentiate specific study contexts to derive practice guidelines from it.

### Modifying design factors

To explain the considerable differences in the effect sizes of the primary studies, various potential moderators were analysed in the meta-analysis. Out of a total of 41 potential moderators investigated [*N* = 21 in Means et al. (2013); *N* = 6 in Bernard et al. (2014); *N* = 6 in Spanjers et al. (2015); *N* = 2 in Vo et al. (2017); and *N* = 6 in Müller and Mildenberger (2021)], very few have turned out to be significant. In contrast to other meta-analyses, Vo et al. (2017) found a significantly higher mean effect size in STEM disciplines compared to that of non-STEM. From an educational design perspective, it is interesting to note that the use of quizzes (or regular tests with feedback for students) has a significant and positive influence on the effectiveness and attractiveness of blended learning (Spanjers et al., 2015).

Bernard et al. (2019) analysed the moderator analysis in more retrospective meta-analyses from 2000 to 2015. They concluded that student interaction, collaboration, and discussion emerged as a moderating influence in several studies. Additionally, practices, feedback, and incremental quizzes (i.e., formative evaluation) also appeared important in several studies. However, they also pointed out that there is a large amount of literature showing that these instructional elements were equally valuable in all educational settings.

The above explanations have shown that the past moderator analyses in meta-analyses could not explain the heterogeneity of the student achievements with the design factors in blended learning, other than confirming that quizzes could enhance effectiveness. Studies based on surveys of students and lecturers—which assess the subjectively perceived learning success and the design factors—can provide further indications for an effective educational design in a blended learning format.

Owston and York (2018) and Hilliard and Stewart (2019) emphasised in their student survey-based studies that regardless of the chosen online or face-to-face ratios, care must be taken when designing a learning environment to integrate interactive and cooperative activities between students as well as between students and instructors. Other studies based on student evaluations (Castaño-Muñoz et al., 2014; Cundell & Sheepy, 2018; McKenna et al., 2020) have also emphasised the importance of student interaction in blended learning. According to Cundell and Sheepy (2018), passive online activities such as videos and readings are not as effective as well-structured activities in which students collaborate with or learn from other students. Content delivery does not equate to a well-designed learning environment or, as Merrill (2018, p. 2) put it, ‘information alone is not instruction’. Thus, students need adequate stimulation, especially in the online part of blended learning (Lai et al., 2016; Manwaring et al., 2017; Pilcher, 2017). Often mentioned is also a thoughtful balance between face-to-face and distance moments (Vanslambrouck et al., 2018). Different instructional strategies were proposed for a blended learning format (McKenna et al., 2020), but these have not been scientifically analysed (except for the flipped classroom, e.g., Müller and Mildenberger (2021)).

In Cundell and Sheepy (2018), peer feedback was also found to be effective for learning; students benefit from analysing the work of others and providing feedback to each other. The importance of feedback in the learning process is well known (Hattie & Timperley, 2007) and has also been shown as a critical design factor in other blended learning studies (Garcia et al., 2014; Martin et al., 2018; Vo et al., 2020).

In addition, other studies also highlight the importance of the social presence of instructors (Goeman et al., 2020; Law et al., 2019; Lowenthal & Snelson, 2017) and the creation of an affective learning climate (Caskurlu et al., 2021; McKenna et al., 2020). These aspects should help reduce social isolation (Gillett-Swan, 2017) in the online part of blended learning. Further studies (Caskurlu et al., 2021; Ellis et al., 2016; Han & Ellis, 2019; Heilporn et al., 2021) have also identified course structure and guidance as important design factors in blended learning.

These last factors, in particular, depend strongly on the teacher’s commitment and understanding of their role. However, implementing a new blended learning format is challenging and time-consuming for instructors and may also provoke resistance (Bruggeman et al., 2021; Huang et al., 2021). Accordingly, plausible motives need to be presented as to why these changes are necessary, and incentives are required to engage lecturers (Andrade & Alden-Rivers, 2019).

Based on the individual studies, the syntheses and reviews (Boelens et al., 2017; McGee & Reis, 2012; Nortvig et al., 2018) come to similar conclusions regarding the key design factors in blended learning. Findings like these indicate which design factors are perceived by students and lecturers as conducive to learning. However, the limitation here is that these factors were surveyed based on subjectively perceived learning success rather than on objectively assessed learning achievement. One such study by Vo et al. (2020) investigated how design factors assessed by students were related to final grades. Of the eight design factors studied, only ‘clear goals and expectations’ and ‘collaborative learning’ were significant predictors of student performance as measured by final grades in different courses. However, the level of final grades measured in various courses may not only depend on performance or instructional design but be influenced by other factors such as the bell-curve tendency of grading (Brookhart et al., 2016), when the grade often represents a student's relative achievement within the whole group (Sadler, 2009). It is, therefore, questionable whether course grades alone can be used as an objective measure to compare the effectiveness of different courses. Accordingly, other factors investigated by Vo et al. (2020), such as instructor feedback, support and facilitation, and face-to-face/online content presentation, may positively affect the quality of the learning environment and student performance; however, they are not adequately captured by comparing grades across courses.

Although research has shown some general patterns across blended learning modalities, the root causes for the learning outcomes in blended learning environments are still not apparent. Graham (2019) suspected the above in the pedagogical practices of blended learning, requiring research to examine more closely what happens at the activity level in blended learning.

## Methodology

### Research context

The Zurich University of Applied Sciences (ZHAW) launched a new flexible learning study programme in a blended learning format (FLEX) in 2015 as part of a comprehensive e-learning strategy (Müller et al., 2018). Its Bachelor’s degree programme in Business Administration is a successful, well-established course of study offered both full-time (FT) and part-time (PT). The FLEX format is, therefore, the third study format for this degree programme.

All Bachelor’s programmes have two levels — the ‘Assessment’ level (60 ECTS credits; two semesters for FT students, three semesters for PT and FLEX students) followed by the ‘Main Study’ level (120 ECTS credits; four semesters for FT students, five semesters for PT and FLEX students) with specialisations in Banking & Finance (B&F) and General Management (GM). For the PT and FLEX formats, a part-time job or family commitment of no more than 60%–70% is recommended. The concept for the new blended learning format was developed in 2014 and tested by running a Business Administration FLEX course. After the pilot course was evaluated and found to be effective (Müller et al., 2018), a total of 44 courses were transformed for the BSc in Business Administration degree programme (2015–2020). The first cohort of FLEX students graduated in 2019.

The main objective of the new blended learning format FLEX was to offer students the best possible opportunities to combine their work and personal responsibilities with a flexible learning study programme. Regarding the number and distribution of classroom sessions over the 14-week term, compatibility with a distant place of residence was the guiding principle. More specifically, the maximum number of overnight stays away from home that would be acceptable to potential students had to be determined. At the same time, regular physical classroom sessions were also considered essential to enable students to reflect on the online content. As a result of these considerations, face-to-face classes for FLEX were reduced by approximately half (51%) compared with the part-time programme and replaced with a virtual self-study phase. This means that FLEX students attended the campus every three weeks for two days and the interjacent asynchronous self-study phase should allow them to learn flexibly. According to the typology of Allen et al. (2007) and the inclusion criteria for the meta-analysis of Müller and Mildenberger (2021), the design can be classified as blended learning. Concerning the dimensions of flexible learning, according to Li and Wong (2018), the FLEX format offered greater flexibility in terms of time, delivery, instructional approach, resources, and support than the conventional study format; however, the format was the same as a traditional course regarding the dimensions content, entry requirement, orientation or goal, and performance assessment.

After the time structure for the new course of study had been determined, the transition to the blended learning design was carried out at the course level. Considering that the design aspects activation, interaction and formative performance assessment have been found in empirical studies to be important for asynchronous online environments, care was taken to ensure that content was not only delivered (using learning videos, learning texts, etc.), but that students elaborated and reflected on it in the virtual self-study phases. In so-called ‘scripting workshops’ (Müller et al., 2018), the content was sequenced, and the educational design was created from scratch (Alammary et al., 2014), according to a defined process using a specially developed didactic visualisation language (see also Molina et al., 2009). Web-based technologies such as LMS Moodle and other tools were used and the content was delivered in digital form, mainly using learning videos produced in-house. Interaction with the teachers during the three-week self-study phases was possible in asynchronous form using the Moodle tools such as forums and chat, but no scheduled online class sessions via video conferencing tools were provided. Table 1 shows key features of the course designs in terms of the number of activities for the design aspects activation, interaction, and formative performance assessment (feedback) in the self-study phases, per course. Since learning videos are an important element of an asynchronous online learning environment and have proven to be effective for learning in the pilot study (Müller et al., 2018) and a recent meta-analysis (Noetel et al., 2021), the number of learning videos per course was also assessed. The number of pedagogical design factors was collected in the LMS Moodle, and the results show the range of the design characteristics in the FLEX implementation for the levels ‘Assessment’ (semesters 1–3) and ‘Main Study’ (semesters 4–8), and overall (semesters 1–8).

**Table 1:** Educational design characteristics of the virtual self-study phases (activities per course)

Study level	‘Assessment’ level (Courses *N* = 80)			‘Main study’ level (Courses *N* = 53)			All courses (Courses *N* = 133)		
	*M*	*SD*	*Range*	*M*	*SD*	*Range*	*M*	*SD*	*Range*
Content delivery									
Learning video	23.6	15.0	0–54	14.9	14.2	0–49	20.2	15.3	0–54
Activation									
Assignments	5.5	8.5	0–31	3.7	4.9	0–15	4.8	7.3	0–31
Interaction									
Forum students	12.2	11.9	0–52	3.3	4.4	0–20	8.6	10.5	0–52
Forum instructors	8.9	10.0	0–45	2.7	4.0	0–18	6.4	8.7	0–45
Performance assessment (formative)									
Quizzes	10.8	11.7	0–40	7.4	7.5	0–30	9.4	10.3	0–40

### Research design

The research design consisted of the cohorts of the experimental FLEX group (B&F cohorts 2015–2019 and GM cohorts 2017–2019, *N* students = 278) with students attending all courses in the new FLEX format and the corresponding cohorts of the control group PT (*N* students = 1068). The FLEX format was implemented in a blended learning design with a reduced classroom teaching time, whereas the PT-learning format was implemented conventionally via classroom teaching. Students of the FLEX and PT cohorts were allocated to classes of 30–60 students each. The number of students (*N*) who started the corresponding study programme in the first semester changed over time because of voluntary dropouts, failed exams, transfers between specialisations, and repeaters.

The gender ratio was almost the same in the experimental FLEX cohort as in the control PT cohort (proportion of female FLEX students = 35%; proportion of female PT students = 36%); however, the average age was slightly higher for FLEX students (24.7 years) compared to PT students (22.2 years). Concerning personality traits, various tests were used to investigate whether students differed regarding teamwork affinity (Lauche et al., 1999), ICT literacy (Kömmetter, 2010), general mental ability (Heller & Perleth, 2000, only cohorts 17), and the competencies of self-study and study organisation and learning-relevant emotions including motivation (Schmied & Hänze, 2016, only cohorts 17). These tests all showed no significant difference between the experimental FLEX group and the PT control group. With the entrance qualification of the vocational baccalaureate, students of a university of applied sciences have similar prior knowledge. To check this assumption, prior knowledge was tested in a pre-test on the topic of business administration for cohort 17 (with specialisations B&F and GM). The questions corresponded to the questions on the topic of business administration in past examinations for the vocational baccalaureate. The results of the pre-test showed no significant differences in prior knowledge between students in the FLEX and PT format in either BF [*t*(94) = 0.619, *p* = 0.537] or the GM [*t*(69) = 0.182, *p* = 0.856] specialisation.

The student eligibility requirements, lecture content, exam questions, and grading scales were identical for all students in the experimental FLEX and the control PT conditions. FLEX students took precisely the same examinations and at the same time as students in the conventional PT programme and the exams were not marked by the class teacher but by an independent pool of lecturers, allowing for a comparison of the exam results with high empirical significance.

### Analysis methods for student achievement

To assess the effectiveness of the blended learning FLEX format, the exam results of the FLEX students (*N* = 2822 exams) were compared with those of PT students (*N* = 11638) in 133 courses between 2015 and 2019 (nine semesters). The effect size (standardised mean difference, also known as Cohen’s *d*) was calculated for each course (i.e., the deviation of the experimental group FLEX test results from the control group PT). A *t*-test for the difference between the two groups (at *α* = 0.05, two-tailed) was performed for each course. Additionally, a test for equivalence with equivalence defined as being between ± 0.5 standard deviations was examined (see also Mueller et al., 2020).

To analyse the overall learning effectiveness of the FLEX study format, the results from each course were aggregated using regression analysis (roughly similar to a meta-analysis). A linear mixed-effects regression analysis was performed with the calculated effect sizes as the response, and potential moderator variables study level, specialisation, and discipline as factors (fixed effects). In addition, a random effect for the cohort was included to control for the dependency arising from the same students attending courses. Assessing the size and significance of the random cohort effect was also of interest. Since good estimates of the standard error of the calculated effect sizes can be calculated from the raw grades, a weighted regression was performed where each effect size was weighted by its inverse estimated variance. This corresponds to the usual weighing scheme in fixed-effect meta-analysis. Using the lme4 package for R (Bates et al., 2020), estimation was performed using restricted maximum likelihood.

### Analysis methods for the modifying factors

An analysis of potential moderating variables that might explain the heterogeneity of the effect sizes was conducted, investigating study level, specialisation of the study programme, disciplines (e.g., quantitative subjects, foreign language, social sciences, or management), and cohorts. As a first step, correlations between various contextual variables (student and lecturer perceptions, educational design characteristics) and the effect sizes of the courses were analysed, and then the critical factors were related to effect size using a multiple linear regression model.

Student perceptions of the new learning design and learning process were analysed through a student course evaluation. At the end of each course, the FLEX group completed a questionnaire consisting of nine items of different instruments—structure, guidance and motivation, coherence (SCEQ), usability (own item), support and learning outcome (HILVE, Rindermann & Amelang, 1994), interest/enjoyment (Intrinsic Motivation Inventory, Ryan, 1982), and two open-ended questions (‘What do you like about the way the course is designed?’, ‘What do you like less?’). Additionally, student attendance in on-campus classes was determined. The surveys took place after the classes had been completed but before the examination period.

Lecturers also rated the implementation conditions with a specially developed 20-item instrument according to the change dimensions in Knoster et al. (2000). This survey took place at the end of the semester when a course was first implemented. Only courses whose instructors were involved in both the development and the implementation of the courses were included in the correlation. Because instructors for individual courses changed in some cases during the test period, a smaller number of courses was analysed than the total number of courses (see Table 6).

The qualitative analysis aimed to discover which factors (especially educational design characteristics) were crucial for the success of a FLEX course. For this purpose, the courses were divided into groups according to their effect size and student evaluation ratings. For the student evaluation criteria (scale 1–5), the courses were divided into three clusters (terciles) with high, medium, and low student ratings. ‘Good practice’ courses were defined as courses with a positive effect size and a high student rating (first tercile). ‘Bad practice’ courses were defined as courses with negative effect size and low student ratings (third tercile). For the qualitative analysis, from a total of 133 FLEX courses, 27 ‘good practice’ courses with a total of 493 student comments (to the question ‘What do you like about the way the course is designed?’), and 30 ‘bad practice’ courses with a total of 429 student comments (to the open-ended questions ‘What do you like less?’ and ‘Do you have ideas on how the course could be developed further?’) were included. These data were imported into MAXQDA, and each student comment was labelled with the study specialisation, semester, student number, course name, and good/bad-practice course designation (e.g., ‘SBF15_HS15_8BWL_good’).

An initial version of a category system was created, which was theory-driven and based on the principles for designing the FLEX courses. The following five categories were defined—educational design (with subcodes: content sequencing, guidance, blend online/classroom-learning), activation (with subcodes: tasks/exercises, cases, solutions), learning resources (with subcodes: textbooks, learning videos), interaction (with subcodes: with peers, with instructor), and performance assessment.

The entire dataset was coded independently by two coders. Because the category system we developed was being applied for the first time, intercoder agreement checks were started after only a few codings in two iterations to identify weaknesses (Kuckartz & Rädiker, 2019). An initial review was based on 10 ‘good practice’ and 10 ‘bad practice’ comments randomly selected from the dataset. A second review took place based on another 15 ‘good practice’ and 15 ‘bad practice’ comments, which were deliberately drawn according to the criterion of completing the theory-based coding guide. In both iteration cycles, the coding was checked for mismatches. The segments where non-matches occurred formed the starting point for a systematic discussion between the two coders about the disagreement, which resulted in an adaptation of the category system and the coding guide (Kuckartz & Rädiker, 2019). Comments that belonged to two subcategories were assigned to the main category.

Next, the two coders independently coded the entire data set. The intercoder agreement was checked at the segment level with a setting of 90% overlap, which resulted in a kappa value of 0.57. One of the coders analysed the mismatched segments and standardised them with reference to the coding guide. The coded segments were then analysed. Initially, a frequency analysis (descriptive counting of code frequency) was conducted by counting the individual codes using MAXQDA. Then, the most important aspects of the respective categories were summarised and provided with appropriate quotations.

## Results

### Student achievement

#### Student achievement at the course level

The FLEX and PT samples were independent, and the sample size and histograms of the test results did not indicate a violation of the requirements of normal distribution and uniformity of variance. The effect size of the students’ exam results (Cohen’s *d*) was calculated by comparing the FLEX courses with the respective PT courses. The direction was indicated by the sign of the effect size (Cohen’s *d*); for example, in 61 of the 133 courses examined, the mean values of the FLEX cohort were higher than those of the PT, corresponding to positive values for the effect size (see Table 2).

**Table 2:** Learning effectiveness of experimental FLEX courses compared with conventional PT courses

Effect size *d*	‘Assessment’ level (Courses *N* = 80)	‘Main Study’ level (Courses *N* = 53)	Total (Courses *N* = 133)
Effect size > 0	42	19	61
*Significant (at α* = *0.05, two-tailed)*	*5*	*5*	*10*
Effect size < 0	38	33	71
*Significant (at α* = *0.05, two-tailed)*	*7*	*7*	*14*
Effect size = 0	0	1	1
*Significant equivalence* *(at α* = *0.05, two-tailed)*	*24*	*12*	*36*
*Inconclusive*	*44*	*29*	*73*

**Fig. 1:**
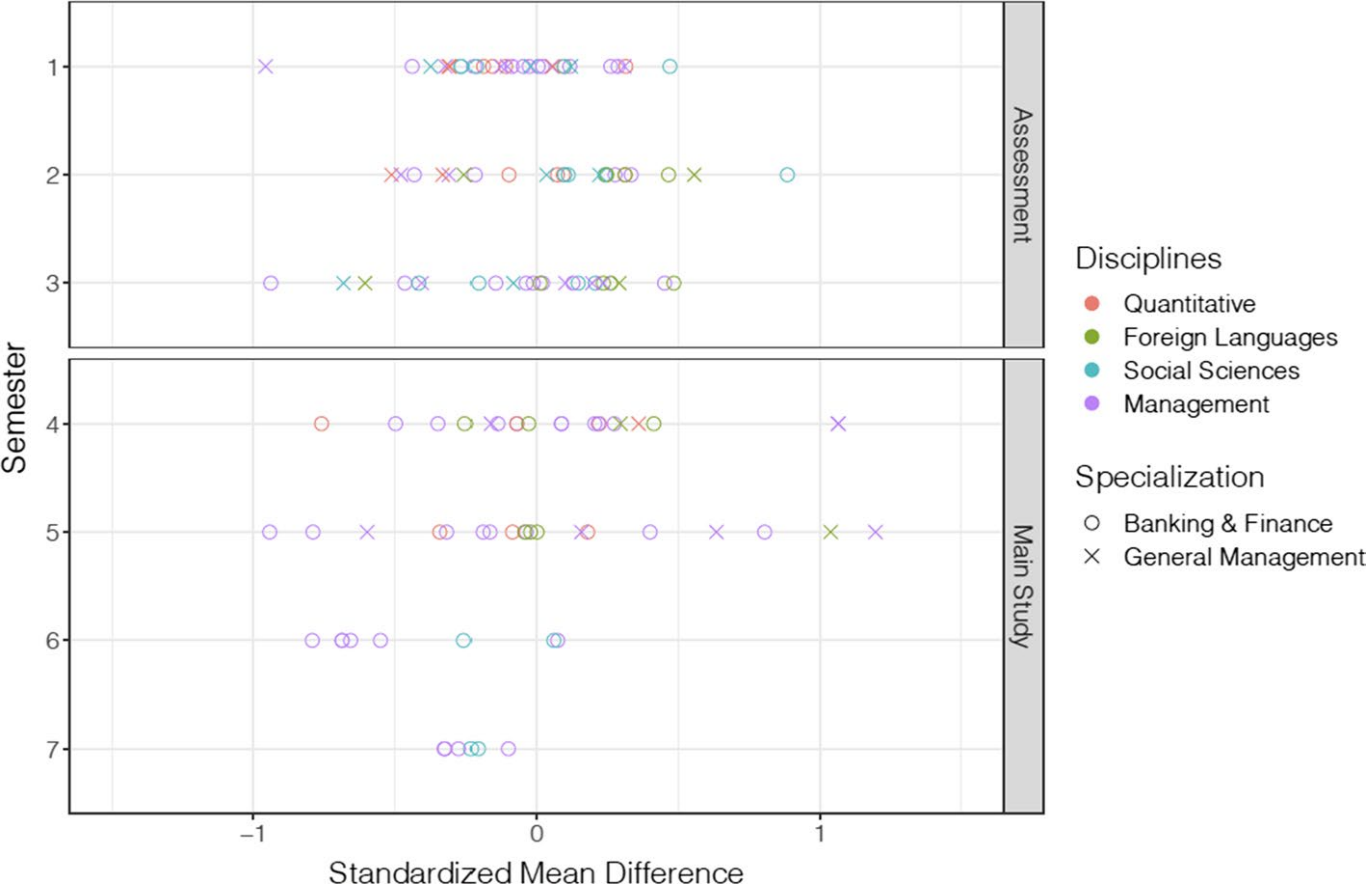
Standardised mean differences (effect sizes) of analysed courses (*N* = 133)

The courses were categorised into four subject groups—quantitative subjects (statistics, mathematics, quantitative methods), foreign language (English), social sciences (law, skills, communication, leadership & ethics), and management (e.g., strategy, accounting, marketing). The distribution of the effect sizes according to the study level, course of study (BF or GM), and subject domain is shown in Fig. 1.

The results for the 133 courses in the ‘Assessment’ and the ‘Main Study’ levels showed that there is little difference in the exam scores of students in the FLEX format compared with the PT format (see Table 2). A *t*-test (*α* = 0.05, two-tailed) indicated a significant difference in only 24 of the 133 courses; FLEX students showed significantly higher exam scores in 10 courses and PT students in 14 courses. To compare FLEX and PT learning performance, it is important to consider that comparative studies usually aim to demonstrate significant change. More precisely, the goal is to reject the H_0_ hypothesis (no differences between groups) and confirm the H_1_ hypothesis (difference between groups exists at a particular significance level). The experimental group (in our case, the FLEX cohort) would, therefore, be expected to perform significantly different from the control group (PT cohort). However, in the research context, this was not a priority. Due to the changed conditions caused by the reduction of classroom time by more than 50 per cent, the goal was instead to ensure that students achieved equivalent exam results with the self-study assignments in the blended learning format compared with the control group, despite the reduction in classroom time. Where the aim is to prove that there are no differences between the results of the two groups, an equivalence test is used. We regard standardised mean differences as equivalent if they are smaller than 0.5 in absolute value, and a statistical equivalence was found in 36 courses. In 73 courses, the difference was inconclusive (no statement possible about statistical difference or equivalence).

#### Student achievement at the programme level

The estimated coefficients of the linear mixed-effects regression analysis can be found in Appendix, Table 6. The estimated summary effect size *d* is close to and not significantly different from zero (see also Table 3). The confidence interval [− 0.206, 0.094] suggests that overall differences between the blended learning format FLEX and the conventional classroom format PT are small and, at best, moderately negative or very small positive effects are plausible. This means that equivalent learning outcomes were found despite a reduction in classroom time for FLEX compared with PT students of over 50 per cent.

**Table 3:** Summary effect size for the mixed-effects regression model

	Effect size and standard error				Confidence interval			*t*-Test	
	*n*	*d*	*SE*	*Lower 95th*		*Upper 95th*	*t*		*p*
Overall effect	133	− 0.0562	0.0562	− 0.2060		0.0936	− 1.0000		0.3684

### Modifying factors

#### Moderator analysis

In Table 4, similar to the moderator analysis in a meta-analysis, the results are presented as group means with corresponding standard errors and 95% confidence intervals. These are not averages of the raw data per group, but calculated from the regression results using the emmeans package for R (Lenth, 2021); for each moderator variable, the other factors were held constant at the proportion in the data set. The overall effect was similarly obtained from the regression estimate, not from averaging the original effect sizes. The significance of the effects of potential moderators was assessed using the Likelihood Ratio Test as implemented in lme4 for R (Bates et al., 2020), with none of the variables having a significant effect.

**Table 4:** Likelihood ratio tests for moderators

Moderators	Effect size and SE			Confidence interval	
	*n*	*d*	*SE*	*Lower 95th*	*Upper 95th*
*Study level, LR* = *0.4422, df* = *1, p* = *0.50607*					
‘Assessment’ level	80	− 0.0330	0.0582	− 0.1697	0.1038
‘Main Study’ level	53	− 0.0913	0.0799	− 0.2710	0.0883
*Specialisation, LR* = *0.4266, df* = *1, p* = *0.51368*					
Banking and Finance	95	− 0.0372	0.0684	− 0.2260	0.1515
General Management	38	− 0.1036	0.0944	− 0.3209	0.1137
*Discipline, LR* = *6.4909, df* = *3, p* = *0.09002*					
Quantitative subjects	21	− 0.1155	0.0876	− 0.2952	0.0642
Foreign language	20	0.1194	0.0952	− 0.0755	0.3143
Social sciences	24	− 0.045	0.0836	− 0.2078	0.1387
Management	68	− 0.0972	0.0638	− 0.2462	0.0518

The significance of the random cohort effect was tested by comparing the full model to a classical linear model including all variables except the cohort effect, again using the Likelihood Ratio Test; this was not significant either (*LR* = 2.098, *df* = 1, *p* = 0.1475). Moreover, the estimated standard deviation for the cohort effect is 0.1186, which is only roughly one-third of the estimated residual standard deviation of 0.3502.

#### Correlation and regression analysis of contextual variables

Although the implementation context of the courses (conceptualisation of blended learning, measurement of learning outcomes, and implementation period of one semester) was quite similar, the effect sizes showed a considerable variance between the courses (see Fig. 1). A correlation analysis was therefore conducted to examine to what extent the student evaluation of the course quality (including attendance rate), the quantitative educational design characteristics, or the survey on the implementation conditions among the lecturers showed a correlation with the effect sizes.

The results of the correlation analysis (Pearson, 2-tailed) indicate the strongest correlation between student course evaluations and effect sizes (see Appendix, Table 7). All items show a significant correlation between student evaluation of course quality and the effect size (e.g., item ‘I like the course’ *r* = 0.289, *p* = 0.001). The course quality assessed by the students, thus, has a significant correlation with the learning effectiveness measured as standardized mean differences between blended and conventional courses. This is remarkable because the course evaluation took place at the time when classes had been completed but before the examination period.

There is also a significant correlation with the reported attendance of the classes; courses whose classroom sessions were attended more frequently show a higher effect size. In contrast, the number of different learning resources and activities in the courses—such as the number of tasks, forum posts, formative quizzes, or learning videos—has no significant correlation with the effect size of the courses.

The correlation between the implementation conditions and the effect size of the courses shows a differentiated picture. For example, the dimensions ‘incentives’ and ‘resources’ do not show a significant correlation with the effect size; however, a significant correlation is reported for the ‘competences’, ‘vision’, ‘action plan’, and ‘satisfaction’ (e.g., item ‘I am satisfied with the introduction of FLEX at the ZHAW’ *r* = 0.303, *p* = 0.013).

A multiple linear regression model was used to evaluate the contribution the data collected from students and lecturers make to the standardised mean difference. Because of substantial correlations between the evaluation variables (‘student course evaluation’ and ‘implementation survey instructors’), the items covering different aspects were averaged to form one aggregated variable for the student evaluation (i.e., ‘student evaluation’) and six aggregated variables for aspects of the instructor evaluation (‘incentives’, ‘resources’, ‘skills’, ‘vision’, ‘action plan’, and ‘satisfaction with the implementation’). To avoid collinearity issues, a stepwise forward procedure was used. Starting from an intercept-only model, all models adding one of the variables were fitted, but only ‘student evaluation’ (*F* = 11.2449, *df* = 1, *p* = 0.0014) and ‘action plan’ were significant (*F* = 7.2867, *df* = 1, *p* = 0090). Starting from a model containing only an intercept and ‘student evaluation’, adding ‘action plan’ did not significantly improve the fit (*F* = 2.3329, *df* = 1, *p* = 0.1320), but adding ‘student evaluation’ to a model that only included ‘action plan’ does (*F* = 5.9408, *df* = 1, 0.0178). In a model including both variables, ‘student evaluation’ is significant (*t* = 2.437, df = 1, *p* = 0.0178) while ‘action plan’ is not (*t* = 1.527, *df* = 1, *p* = 0.1320). The optimal model was obtained by the forward selection, containing only an intercept and ‘student evaluation’, although the adjusted R-squared value is not high (0.1438). For this reason, the results are not reported here in detail.

### Qualitative analysis of educational design quality

The frequency of coded student comments on educational design quality is reported in Table 5. The student comments contained a vast number of mentions related to educational design in both the ‘good practice’ and the ‘bad practice’ courses (60.4% and 50.0% of all mentions). Within this category, it is also noticeable that many comments referred to the blending of online and classroom components (20.9% and 28.6%). Furthermore, many comments addressed the guidance provided (10.6% and 9.3%). There were a similar number of mentions in the learning videos subcategory (9.9% and 9.3%). Noticeably fewer mentions were related to the textbook/other texts (6.2% and 10.5%), assignments (6.2% and 6.5%), and performance assessment (6.2% and 6.0%). In the case of the ‘bad practice’ courses, the subcategory solutions also stand out (8.9%). There were a very low number of mentions related to interaction with peers (0.4% and 1.2%).

**Table 5:** Frequency of student comments for each category/subcategory

Category and subcategory	Good practice		Bad practice	
	*N*	*In %*	*N*	*In %*
*Educational design*	*68*	*24.9*	*26*	*10.5*
Guidance	29	10.6	23	9.3
Content sequencing	11	4.0	4	1.6
Blending of the online and classroom sessions	57	20.9	71	28.6
*Subtotal educational design*	*165*	*60.4*	*124*	*50.0*
*Content delivery/learning resources*	*4*	*1.5*	*6*	*2.4*
Textbooks	17	6.2	26	10.5
Learning videos	27	9.9	23	9.3
*Subtotal content delivery/learning resources*	*48*	*17.6*	*55*	*22.2*
*Activation*	*1*	*0.4*	*0*	*0.0*
Assignments	17	6.2	16	6.5
Cases	1	0.4	6	2.4
Solutions	1	0.4	22	8.9
*Subtotal activation*	*20*	*7.3*	*44*	*17.7*
*Interaction*	*4*	*1.5*	*0*	*0.0*
With peers	1	0.4	3	1.2
With instructor	18	6.6	7	2.8
*Subtotal interaction*	*23*	*8.4*	*10*	*4.0*
*Performance assessment*	*17*	*6.2*	*15*	*6.0*
Total comments	273	100.0	248	100.0

Student comments indicate that an adequate structure and guidance are essential for the quality of the FLEX blended learning courses. The structure is described as the clear distinctness of topics and their logical sequencing as follows: ‘*The exact structuring of the topics’* (SBF15_HS15_8BWL_good) and ‘*better delimitation and structuring of individual topics’* (SGM17_FS18_1FAC_bad). As guidance, the focus concerning exam relevance in the classroom course is mentioned as *‘The content is clearly linked to the exams, and it is clear what is expected’* (SBF17_HS17_9MAR_good). This aspect also includes the desire for mock exams or the availability of exams from previous years. In addition, guidance is described as a review and outlook by the lecturers and the indication of the learning progress in the learning management system.

The subcategory ‘blending’ contains the appropriate combination of the online and classroom phase(s) (and vice-versa). This link can be achieved by taking up and deepening certain content from the online phase in the classroom or by linking to it and continuing with it. A diverging picture emerges concerning the design of the classroom phase. While some students would have liked to repeat the content from the online phase and set a focus, others would have preferred to consolidate and deepen the content from the online phase through exercises and discussions. The following statements well illustrate this divide: *‘I did not like the fact that some students came to the lectures unprepared and asked basic questions. In this way, the other students did not benefit. […] I talked to many students, and many of them had done very little preparation before the lecture and then asked many questions in the lecture. That really doesn’t work, in my opinion’.* (SBF17_HS17_19MAT_bad); *‘More complex topics are treated in the classroom phase’.* (SGM17_HS17_1MAR_good); *‘Teaching could be more efficient. It cannot be assumed that all FLEX students have solved everything that is on Moodle [tasks on the Learning Management System]. A misconception’.* (SBF15_FS17_7MAC_bad); and *‘Repetition of the material learned in the online phase’.* (SGM17_HS17_14MAR_good).

The following student statements also raise the question of optimal allocation of scarce classroom time: *‘The lecturer asks few questions and delivers many monologues. For that, I could actually watch a video instead’.* (SGM19_HS19_10WIR_bad) and ‘*The way the classroom sessions are structured is good. At the beginning, a short repetition of the theory and then working on tasks. This helps us to repeat and apply all the material learned’.* (SGM18_HS19_3MIK_good).

In the category ‘content delivery’, the compactness of the learning resources and their alignment with the online weeks was mentioned. The linking of instructional texts, PowerPoint slides, and learning videos was brought up in the context of learning resources. In the case of instructional texts, students mentioned their comprehensibility and, in the case of learning videos, their existence, quality, and adequate length: *‘Good structure with linking of book, slides, and videos’.* (SBF19_HS19_19MAR_good).

In the ‘activation’ category, the number and variety of exercises and their consistency with the theory learned were mentioned. In addition, the existence of solutions to tasks and exercises was cited as crucial for the online phase in three respects—the solutions must be complete (i.e., solutions to all tasks), sufficiently detailed (i.e., with solution path included), and readily available (i.e., at the time when students solve the tasks); *‘Not having a complete solution script inhibits the learning process very much if I always have to ask for the solution in the forum every time I have [already] finished an assignment. When then the answer finally comes, I am already somewhere else again—very counterproductive’!* (SBF16_HS17_14MIK_bad).

In the ‘interaction’ category, the opportunity to ask questions and get a quick answer from the lecturers was frequently mentioned for both the classroom and the online phases. A well-maintained forum (opportunity to place questions in the LMS system) was also mentioned for the online phase. Although there were few comments about peer interaction, it was noticeable that group work was seriously questioned: *‘In general, the obligation to participate in group performance assessments is paradoxical and pointless in the context of the goals of this part-time FLEX course’.* (SBF15_FS17_19EBF_bad).

In the ‘performance assessment’ category, formative tests with automatic and immediate feedback were mentioned: *‘I also like the small exams for self-testing because you can check what you have understood’.* (SGM18_HS18_1WIR_good).

## Discussion and conclusions

Results from the first research question demonstrated that the estimated effect size for a flexible learning study programme in a blended learning design with a 51% reduced on-site classroom time was close to and not significantly different from zero. This result is in line with previous studies (e.g., Müller & Mildenberger, 2021), suggesting that a blended learning format with reduced classroom time is not systematically more or less effective than a conventional study format. This study also indirectly confirmed the recommendations of various authors (Hilliard & Stewart, 2019; Owston & York, 2018) to divide the online and face-to-face portions of blended learning in half. Similar to the results of other studies and reviews on blended learning (Bernard et al., 2014; Means et al., 2013; Müller & Mildenberger, 2021; Spanjers et al., 2015; Vo et al., 2017), the effect sizes of the courses were broadly scattered around zero, with almost one standard deviation in the minus to over one standard deviation in the plus.

Findings from the second research question addressed the modifying factors for the learning effectiveness of blended learning courses with reduced classroom time. The analysed moderators of ‘study level’, ‘specialisation’, and ‘disciplines’ can be classified as moderating effects of condition (Means et al., 2013). The non-significant results for the study level are in line with the findings of systematic reviews by Bernard et al. (2014) and Means et al. (2013), who found no moderation effects on the course level (undergraduate vs graduate course). The non-significant result of the moderator ‘discipline’ corroborates the systematic reviews of Müller and Mildenberger (2021) and Bernard et al. (2014). However, it is not in line with Vo et al. (2017), who found a significantly higher effect size for STEM disciplines. Different definitions of these disciplines may explain the differences in these findings.

Based on the results of this study and the systematic reviews conducted in the past, it can be concluded that the heterogeneity of the results is not likely to be attributable to conditional factors such as the study level or discipline. However, significant correlations were reported between the effect sizes of the courses and the educational quality and design evaluated by students, the implementation conditions evaluated by lecturers, and on-site class attendance. There is collinearity between these aspects, and it can be assumed that there is a causal relationship in the sense that on-site attendance is influenced by the educational design and the quality of the course. Furthermore, the latter, in turn, is impacted by the attitude and motivation of the lecturers towards the FLEX programme. However, apart from the educational quality as evaluated by the students, significant direct and indirect effects could not be established with the fitted multiple linear regression model.

The importance of the educational design for the effectiveness of blended learning was supported by the significant moderator analyses of Spanjers et al. (2015) regarding the use of quizzes. In contrast, no correlation was shown between the number of online learning resources and activities in the courses, such as the number of assignments, forum posts, formative quizzes, or learning videos, on the one hand, and the effect sizes, on the other. This indicates that educational quality goes beyond the mere number of activities or particular learning resources and that an appropriate educational design is decisive (Graham, 2019; Nortvig et al., 2018).

The qualitative design analysis of the courses with high vs low learning effectiveness identified several crucial design factors for learning-effective blended courses. Regarding educational design, an adequate course structure and guidance for students are recognised as essential. In the context of an undergraduate programme, this means, in particular, that the learning environment has a clear structure, and that sufficient guidance is provided. This factor is significant in blended learning because the combination of online and face-to-face teaching and the partial distance between teachers and students increase the complexity of the learning environment. In this regard, a thoughtful alignment of the online and on-site learning phases was also mentioned; however, the feedback was contradictory concerning the instructional strategy (McKenna et al., 2020). While some students prefer to consolidate and deepen the content from the online phase through exercises and discussions, others simply prefer to repeat it. Such feedback must be seen in the context of the flexible learning study programme FLEX, which offers students opportunities to combine their work and personal responsibilities with study and, therefore, possibly attracts students who place a high priority on pedagogic efficiency. The delicate balance between work, private life, and education is, therefore, more keenly felt by these students and could result in insufficient time to complete all the online tasks. Consequently, guidance also means that instructors should explain how the online and on-site phases are integrated and help their students understand that the online environment is an essential part of the blended learning experience (see also Ellis et al., 2016; Han & Ellis, 2019).

Regarding content delivery, good practice is characterised by learning resources that are well linked and aligned with other elements, such as the tasks in the learning environments. In line with the pilot study (Müller et al., 2018) and a recent systematic review (Noetel et al., 2021), learning videos are appreciated by students and considered to have many educational benefits.

The relevance of activation was also pointed out in the qualitative analysis. These learning activities enable students to transform the information they have acquired into knowledge and skills and facilitate their ability to apply learned knowledge and skills in new and real-life situations. In addition to previous studies (Cundell & Sheepy, 2018; Lai et al., 2016; Manwaring et al., 2017; Pilcher, 2017), the instant availability of complete and detailed solutions when students learn with tasks and exercises is essential for the learning process and its effectiveness.

Regarding the aspects of interaction and assessment, the results corroborate previous studies as the good practice is associated with the social presence of instructors and their prompt feedback (Goeman et al., 2020; Law et al., 2019; Lowenthal & Snelson, 2017) and the availability of formative tests with immediate, often automatic feedback (Garcia et al., 2014; Martin et al., 2018). At the same time, the interaction between students is controversial, and group work is questioned. This may result from the previously discussed need for efficiency in a flexible learning study programme. However, other studies (Gillett-Swan, 2017; Vanslambrouck et al., 2018; Vo et al., 2020) have also pointed out that a blended learning design may also be associated with specific costs, such as the practical issue of organising group work.

### Theoretical and practical implications

The presented work in this study has theoretical and practical contributions and implications. Theoretically, this study expanded the database regarding the learning effectiveness of blended learning with reduced attendance time in several ways and provides important findings. First, past studies on blended learning with reduced classroom time were, with a few exceptions (e.g. Chingos et al., 2017), designed as single studies with a limited duration of usually one semester (Müller & Mildenberger, 2021). In contrast, this study extended these findings at the study programme level encompassing many courses (133 courses) in different disciplines over more than four years (nine semesters). Additionally, it was not designed as a model project with privileged conditions such as selected lecturers and additional resources but introduced using existing equipment and regular teaching staff. Accordingly, a high ecological validity can be assumed.

Similar to the meta-analyses on blended learning (Bernard et al., 2014; Means et al., 2013; Müller & Mildenberger, 2021; Vo et al., 2017), the observed variance in the learning effectiveness of the individual courses was large. The findings of this study demonstrated that the heterogeneity of the effect sizes could be explained by differences in the implementation quality of the educational design factors. This study is the first we are aware of that investigated design factors based on the relative effect sizes of individual courses and not only on student and lecturer evaluation.

The results of this study provide institutions and administrators with practical guidance for their flexible learning initiatives, especially concerning learning effectiveness and the related design principles of a flexible learning programme in a blended learning format. Based on our findings, we recommend paying particular attention to the following educational design principles when implementing blended learning courses:

1. Adequate course structure and guidance for students.
2. Activating learning tasks.
3. Stimulating interaction and social presence of teachers.
4. Timely feedback on the learning process and outcomes.

Instructors are responsible for designing and implementing these factors, and this study showed that the quality of the educational design was significantly related to lecturer attitudes towards blended learning with reduced on-site classroom time. Accordingly, when introducing blended learning to an educational institution, it is vital not only to provide the necessary infrastructure and resources and develop the skills needed to teach a blended learning format but also to provide lecturers with incentives for engagement. At the same time, a shared vision of a flexible learning environment in a blended learning design should be developed to initiate and establish a new learning culture.

Finally, the student evaluation of the course quality has a significant correlation with the relative effect sizes of the individual courses. Thus, students seem to have a good sense of what blended learning conditions they require to succeed. Accordingly, we recommend educational institutions actively involve students in developing blended learning designs, even to the extent of forming pedagogical partnerships (Cook-Sather et al., 2019).

### Limitations and future directions

The design of this study was strictly controlled for a field study in an educational area. Due to identical learning objectives and exams, the framework conditions of the two study formats were comparable, the presence of a control group ensured a quasi-experimental design, and selection bias was controlled. Additionally, as this study was not carried out in a model project with unique resources, support, and incentives, a high ecological validity can be assumed in an authentic university setting with regular lecturers. Nevertheless, the study is subject to the inherent limitations of a real-life setting.

Concerning the data set, because the university had to switch from a mainly on-site format to exclusively hybrid and online formats during the COVID-19 pandemic, cohorts could be surveyed at different study levels, and only one complete cohort could be observed, uninterrupted, from entry to graduation. Accordingly, relatively few courses from the upper semesters of the ‘Main Study’ level were included compared to ‘Assessment’ level courses.

Another limitation of this study is that the flexible learning study programme in a blended learning design we analysed appeals necessarily to a particular student population, namely those with limited time and/or a greater need for spatial flexibility, often because of a demanding job or family commitments. As a result, although the FLEX and PT groups were similar in terms of the control variables and the pre-test, bias due to self-selection could not be ruled out. It should, therefore, be acknowledged that the results concerning the blended learning format are of limited generalizability beyond a context of a flexible learning study programme. It was also shown that the needs of students regarding flexible learning programmes can be highly specific. Therefore, in the future, it would be essential to differentiate research on the design of blended learning depending on the particular study context.

Furthermore, this study identified design factors for blended learning courses based on the relative effect sizes of individual courses. Future studies should verify and differentiate the results of this study to arrive at validated practice guidelines.

## Conclusions

This work contributes to the growing literature on the implementation of flexible learning study programs in a blended learning design. Overall, this study found equivalent overall learning effectiveness in a blended learning format with reduced classroom time by 51% compared with the conventional study format. The study provides evidence that making education more flexible by offering blended learning with reduced classroom time can improve access to education without compromising learning effectiveness. Additionally, the learning effectiveness of the individual courses was found to be moderated by the implementation quality of the educational design factors. Specifically, an adequate course structure and guidance for students, activating learning tasks, stimulating interaction and social presence of teachers, as well as timely feedback on the learning process and outcomes, were identified as crucial design principles for learning-effective blended learning courses.

The results encourage higher education institutions to offer flexible study programmes in a blended learning format with reduced classroom time but also underscore the importance of the educational design quality.

## Data Availability

The datasets are available from the corresponding author on reasonable request.

## Appendix

See Tables 6, 7.

**Table 6:** Regression coefficients

Fixed effect	Est. coefficient	Std. error	t value
(Intercept)	− 0.07326	0.09821	− 0.746
Study Level: “Main Study” vs “Assessment” level	− 0.05836	0.07463	− 0.782
Specialization: GM vs BF	− 0.06639	0.11175	− 0.594
Discipline: Foreign Languages vs Quantitative	0.23490	0.11019	2.132
Discipline: Social Sciences vs Quantitative	0.08094	0.09946	0.814
Discipline: Management vs Quantitative	0.01827	0.08626	0.212

Random effect	Variance	Standard deviation
Cohort (Intercept)	0.01406	0.1186
Residual	0.12266	0.3502

**Table 7:** Correlation analysis effect sizes for FLEX courses

	Pearson-correlation	Sig (2-tailed)	*N*
*Student course evaluation*			
Overall student course evaluation	*0.328***	*0.000*	*123*
* Structure*: The content structure of the module is logical and comprehensible to me	0.251**	0.005	123
* Guidance*: It is usually clear to me where I stand and what is expected of me	0.244**	0.007	123
* Support*: There is good support during the self-study phase	0.344**	0.000	123
* Motivation*: I find the self-study phase motivating	0.313**	0.000	123
* Motivation*: I find the classroom sessions motivating	0.307**	0.002	102
* Learning Outcome*s: I learn a lot in the self-study phase	0.180*	0.047	123
* Learning Outcomes*: I learn a lot in the classroom sessions	0.312**	0.001	102
* Coherence*: The learning activities in the self-study phase are well aligned with the classroom sessions	0.349**	0.000	123
* Interest/enjoyment*: I like the course	0.289**	0.001	123
Attendance	0.261**	0.004	119
*Educational design characteristics (numerical)*			
Learning videos	− 0.111	0.205	132
Assignments	− 0.017	0.845	133
Forum students	− 0.007	0.939	132
Forum instructors	− 0.021	0.810	131
Quizzes	− 0.050	0.570	132
*Implementation survey instructors*			
* Incentives total*	*0.225*	*0.065*	*68*
Developing teaching quality is of great concern to me	0.115	0.350	68
Compensation (in hours) for developing and delivering FLEX courses is appropriate	− 0.081	0.511	68
My engagement in developing and delivering FLEX classes is rewarded in other ways besides compensation (in hours) at the ZHAW	0.178	0.147	68
* Resources*	*0.155*	*0.207*	*68*
The information technologies available (Moodle, etc.) and related support were adequate for my needs	0.053	0.666	68
Sufficient time was available to develop the FLEX module	0.131	0.285	68
During the development and implementation of the FLEX module, I was effectively guided and supported as needed	0.197	0.125	62
The introductory/continuing education sessions on FLEX were valuable for the development and implementation of the FLEX learning environment	0.240*	0.049	68
* Competencies*	*0.130*	*0.292*	*68*
I feel able to realise my didactic ideas with Moodle and other e-learning tools	− 0.044	0.724	68
I feel able to develop good learning resources (e.g., learning videos, etc.) for the online self-study phase	0.335**	0.005	68
I feel able to design and manage the student learning process in FLEX effectively	0.227	0.062	68
I feel able to guide FLEX students well in the self-study phase and provide feedback	0.205	0.094	68
I feel able to anticipate the student learning process in a FLEX learning environment and adjust the didactic-methodical design accordingly	0.290*	0.017	67
* Vision*	*0.197*	*0.116*	*65*
The implementation of FLEX is compatible with the desired learning culture at the ZHAW	0.345**	0.006	61
There is a consensus among the lecturers regarding FLEX goals and didactic implementation	0.273*	0.033	61
The school’s executive committee supports the introduction of FLEX	0.406**	0.001	67
* Action plan*	*0.349***	*0.004*	*67*
The introduction of FLEX is organised and managed well	0.333**	0.006	67
The timeline for the introduction of FLEX was good	0.439**	0.000	67
There were sufficient opportunities for the faculty to discuss and reflect on the experience of developing and implementing FLEX courses	0.327**	0.007	67
* Satisfaction*	*0.289**	*0.018*	*67*
I am satisfied with the development and implementation of my FLEX course	0.303*	0.013	67
I am satisfied with the introduction of FLEX at the ZHAW	0.303*	0.013	67

**p* < 0.05. ***p* < 0.01

## Abbreviations

B&F: Banking & Finance
COVID-19: Coronavirus disease 2019
FLEX: Flexible learning study programme
FT: Full-time study programme
GM: General management
LMS: Learning management system
PT: Part-time study programme
STEM: Science, Technology, Engineering, and Mathematics (subjects)
